# Quantifying the impact of rising food prices on child mortality in India: a cross-district statistical analysis of the District Level Household Survey

**DOI:** 10.1093/ije/dyv359

**Published:** 2016-04-10

**Authors:** Jasmine Fledderjohann, Sukumar Vellakkal, Zaky Khan, Shah Ebrahim, David Stuckler

**Affiliations:** ^1^ Department of Sociology, University of Oxford, Oxford, UK,; ^2^ Public Health Foundation of India, Delhi, India and; ^3^ Department of Non-Communicable Disease Epidemiology, London School of Hygiene and Tropical Medicine, London, UK

**Keywords:** food prices, mortality, child health, India

## Abstract

**Background:** Rates of child malnutrition and mortality in India remain high. We tested the hypothesis that rising food prices are contributing to India’s slow progress in improving childhood survival.

**Methods** : Using rounds 2 and 3 (2002—08) of the Indian District Level Household Survey, we calculated neonatal, infant and under-five mortality rates in 364 districts, and merged these with district-level food price data from the National Sample Survey Office. Multivariate models were estimated, stratified into 27 less deprived states and territories and 8 deprived states (‘Empowered Action Groups’).

**Results** : Between 2002 and 2008, the real price of food in India rose by 11.7%. A 1% increase in total food prices was associated with a 0.49% increase in neonatal (95% confidence interval (CI): 0.13% to 0.85%), but not infant or under-five mortality rates. Disaggregating by type of food and level of deprivation, in the eight deprived states, we found an elevation in neonatal mortality rates of 0.33% for each 1% increase in the price of meat (95% CI: 0.06% to 0.60%) and 0.10% for a 1% increase in dairy (95% CI: 0.01% to 0.20%). We also detected an adverse association of the price of dairy with infant (b = 0.09%; 95% CI: 0.01% to 0.16%) and under-five mortality rates (b = 0.10%; 95% CI: 0.03% to 0.17%). These associations were not detected in less deprived states and territories.

**Conclusions:** Rising food prices, particularly of high-protein meat and dairy products, were associated with worse child mortality outcomes. These adverse associations were concentrated in the most deprived states.

Key Messages

- Food prices rose substantially in India between 2002 and 2008.
- Rising food prices, particularly high-protein items, are associated with higher child mortality risks.
- Adverse associations of food prices with child mortality are concentrated in India’s most deprived states.

## Introduction

India achieved tremendous economic growth over the past several decades, becoming the world’s third largest economy, ^1^ but its child and infant mortality rates rank among those of the worst 50 nations. ^2^ Although under-five mortality fell from 145 deaths per 1000 live births in 1985–90 to 130 per 1000 in 2005–10, ^3^ marking a substantial reduction, India is not on track to achieve the Millennium Development Goal to reduce under-five mortality by two-thirds by 2015. ^4^ This high burden of child mortality occurs in the context of very high undernutrition. ^5^ It is estimated that one in every three malnourished children globally lives in India, where 47% of children under age three are underweight, 46% are stunted and 16% are wasted. ^6^ According to the latest available data from 2005, about one-third of all deaths among children under five were attributable to low birthweight or prematurity, and a further 30% were attributable to diarrhoeal diseases. ^7^

Food prices have been rising faster than the rate of economic growth, decreasing food affordability and potentially stymying nutritional progress. ^8^ Real food prices increased by 11.7% between 2002 and 2008 (see Supplementary Figure 1 , available as Supplementary data at *IJE* online). This increase may reduce overall calorie intake, worsen the quality of diets and decrease dietary diversity. ^8^ Each of these factors has been linked to elevated risks of stunting, undernutrition and wasting, ^12^ which are in turn strongly associated with child mortality cross-nationally. ^13–16^ Additionally, as prices rise, increasing the share of income required to sustain food intake, food expenditures may crowd out spending on other health determinants, such as children’s clothing, schooling and healthcare. ^17–21^ Maternal nutrition is also likely to deteriorate, increasing risks of low birthweight. ^22^ Although some households may develop resilience strategies, women and children living in households which are already food insecure may be tipped into acute malnutrition and food shortage.

Although numerous studies document the association between food prices and consumption of processed, high-fat foods, particularly animal fats and dairy, ^23–26^ there is a dearth of research evaluating their impact on malnutrition and child survival. The few existing studies on this topic are largely based on simulations rather than empirical analyses ^8^^,^^27^ or focus on aggregated national trends, ^19^ ignoring subnational trends that are likely tied to localized food prices. In low-income countries, reductions in child mortality are slower for the poor than the wealthy, and slower in rural vs urban areas ^28^ —phenomena which state- or national-level studies likely miss. Whereas there is mounting evidence that important variation in mortality rates can be found below the state level, ^29^ no district-level empirical analysis of the relationship between food prices and child mortality in India has been conducted.

Here, we test the hypothesis that rising food prices are a significant contributing factor to children’s mortality risks in India, with worse consequences in the most deprived states and territories. We link rural food price data from the National Sample Survey Office (NSSO) with child mortality estimates from the Indian District Level Household Survey (DLHS) for 2002-08, the latest years for which data were available.

## Methods

Data were collected from three sources: India's cross-sectional, nationally representative DLHS, ^30^^,^^31^ the NSSO rural price data (a misnomer, as urban districts are also included) ^32^ and the Central Statistical Organisation of India. ^33^ Mortality and socio-demographic data came from rounds 2 (2002–04) and 3 (2007–08) of the DLHS. In round 2, data were collected for 507 622 currently married women aged 15–44; unmarried women were not sampled in this round. Of these, 57 324 had never given birth and were excluded. Data on birth and death histories for children born in the 59 months preceding the survey were used to calculate mortality rates, resulting in a final sample size of 248 843 women and 367 023 children. Round 3 included data for 643 945 women, including ever-married women aged 15–49 and never-married women aged 15–24. For consistency with round 2 we dropped never-married women. Of the 643 945 women, 428 004 had not given birth since 1 January 2004, and were excluded. The final sample for round 3 included 215 941 women and their 284 087 children.

Mortality rates were calculated based on mother’s report of the child’s age in months and, where applicable, age at death for all children. Using Stata’s *ltable* function, life tables were constructed to estimate mortality for neonates (0–30 days), infants (0–12 months) and under-fives (0–59 months). These figures were calculated for each of the 601 districts on the DLHS data, and were weighted to the DLHS district weight. District-specific weighted means were calculated for socio-demographic controls including mean parity, religion (proportion Hindu or Muslim), place of residence (proportion urban), household wealth tertile (proportion in low and middle tertiles; since India’s economy is largely informal, the DLHS constructs wealth tertiles based on an index of ownership of household goods and does not collect wage data) and caste (proportion scheduled caste, scheduled tribe or other backwards class). As a proxy for the uptake and quality of the healthcare system, district-weighted means were also included for the proportion of children who received all recommended doses of the diphtheria, pertussis and tetanus (DPT) vaccine and the mean number of antenatal care visits mothers reported for their most recent birth. Given the association between child health and women’s education found in previous literature, ^34^ we also controlled for the mean years of women's education.

These district mortality and socio-demographic data were linked with five rounds of price data for over 130 food items from the NSSO Rural Hub data, corresponding to the DLHS survey dates. Price data were collected from a randomly selected sample of local market hubs across India. To adjust for the possibility that high prices correlate with higher incomes, all prices were adjusted for inflation using the consumer price index for non-food items. Annual prices for each food item were calculated and aggregated into 10 categories: all food items, cereals, pulses, meat, fish, poultry, dairy, vegetables, fruit and sugar. These NSSO price data were then linked to the DLHS mortality data for 389 districts. Since the NSSO was sampled randomly, exclusion of unmatched districts would be unlikely to generate systematic bias with regard to our study questions. Based on the leverage of individual outliers calculated from avplots, we dropped 25 cases with the majority dropped on mortality rates, reflecting sometimes insufficient numbers of deaths at the district level. Data on gross state domestic product (SDP), in constant 1999–2000 rupees, came from the Central Statistical Organisation of India. ^33^ Data were unavailable for Daman and Diu, Dadra and Nagar Haveli, and Lakshadweep. The final sample size was 364 districts.

### Statistical models

We applied first-differenced linear regression models to test the relationship between food prices and mortality.

(1) ΔMortalityi=α+βΔfood pricei+βΔSDP+βpovertyi+βdemographicsi+βhealthcarei+εi

Here, *i* is the district and Δ represents percentage change between waves 2 and 3. Child mortality outcomes included the change in the neonatal (NNMR), infant (IMR) and under-five (U5MR) mortality rates. Δ food prices represents the percentage change in food prices across each of the food items outlined above, modelled separately for each food item. Δ SDP is the percentage change in the state domestic product between waves 2 and 3. SDP is measured at the state level, which may mask district-level heterogeneity; we included both a static measure, which captures the resources available in the state, and a change score measure, which reflects the availability of new funds (or, conversely, a reduction in available resources) to capture variability between districts across time. Static district level socio-demographic controls were aggregated from wave 3 of the DLHS data. Change scores were not used for these indicators as there was relatively little change over time and the absolute level, rather than the change, influences child mortality risk more. Poverty included the proportion of households in the low or middle wealth tertiles, as well as the log of the state domestic product. Demographics is a vector of controls including the mean maternal age, parity and education, and the proportions in urban areas, Hindu or Muslim, and in scheduled tribes, scheduled castes or other backward classes. Health care includes the proportion of children who received all DPT immunizations and the mean number of antenatal care (ANC) visits. Bivariate associations between predictors and dependent variables are provided in Table 1 . We estimated models separately for NNMR, IMR and CMR for each food item. We then stratified states based on whether the Indian government identifies them as economically deprived or not (Empowered Action Group states, hereafter EAG states).

**Table 1.** Bivariate associations of % change in food prices and sociodemographic controls, DLHS

	Full Sample			Non-EAG States			EAG States		
	NNMR	IMR	U5MR	NNMR	IMR	U5MR	NNMR	IMR	U5MR
% Change in all food prices	0.27	0.26	0.36*	0.11	0.11	0.24	−0.02	−0.07	0.21
	(0.15)	(0.24)	(0.14)	(0.15)	(0.25)	(0.13)	(0.16)	(0.25)	(0.12)
% Change in cereal prices	−0.05	−0.04	−0.07	0.00	0.01	−0.05	0.01	0.03	−0.05
	(0.07)	(0.09)	(0.09)	(0.07)	(0.10)	(0.08)	(0.07)	(0.10)	(0.07)
% Change in pulse prices	−0.56*	−0.86*	0.09	−0.70**	−1.06**	0.11	−0.79**	−1.18**	0.10
	(0.25)	(0.37)	(0.27)	(0.26)	(0.39)	(0.24)	(0.26)	(0.39)	(0.22)
% Change in meat prices	0.07	−0.21	0.36*	0.04	−0.24	0.31*	0.24	0.08	0.30*
	(0.15)	(0.25)	(0.15)	(0.15)	(0.24)	(0.13)	(0.15)	(0.24)	(0.12)
% Change in fish prices	−0.02	−0.04	0.00	−0.05	−0.11	0.02	−0.14	−0.30	0.04
	(0.11)	(0.20)	(0.10)	(0.11)	(0.19)	(0.09)	(0.12)	(0.21)	(0.08)
% Change in poultry prices	−0.05	−0.06	−0.06	−0.08	−0.11	−0.07	−0.05	−0.10	−0.03
	(0.10)	(0.16)	(0.09)	(0.10)	(0.17)	(0.08)	(0.10)	(0.17)	(0.07)
% Change in dairy prices	0.03	−0.02	0.10**	0.02	−0.04	0.08*	0.01	−0.07	0.09**
	(0.04)	(0.07)	(0.04)	(0.04)	(0.08)	(0.03)	(0.05)	(0.08)	(0.03)
% Change in vegetable prices	0.11	−0.03	0.38**	0.24	0.43	0.22	−0.10	−0.19	0.20
	(0.16)	(0.28)	(0.13)	(0.16)	(0.28)	(0.12)	(0.16)	(0.28)	(0.11)
% Change in fruit prices	−0.02	−0.01	−0.07	0.02	0.03	−0.01	0.02	0.02	0.01
	(0.08)	(0.12)	(0.09)	(0.08)	(0.12)	(0.08)	(0.08)	(0.12)	(0.08)
% Change in sugar prices	0.09	0.06	0.04	−0.07	−0.19	−0.06	−0.18	−0.41	−0.03
	(0.24)	(0.39)	(0.22)	(0.24)	(0.40)	(0.19)	(0.25)	(0.41)	(0.18)
Proportion of Hindu HHs in district	−60.50***	−63.50**	−37.00	−74.6***	−78.1***	−26.50	−55.00***	−53.2**	−10.20
	(13.70)	(19.70)	(21.00)	(13.30)	(19.30)	(18.70)	(13.70)	(20.10)	(17.00)
Proportion of Muslim HHs in district	117.40***	140.4***	27.30	108.4***	124.6***	30.00	71.00***	79.2**	14.90
	(19.20)	(26.80)	(25.10)	(19.80)	(28.30)	(22.20)	(20.50)	(29.70)	(20.20)
Proportion scheduled caste HHs in district	−19.70	−25.60	−28.90	−39.20	−70.00	6.41	−52.20	−87.80	3.72
	(31.50)	(46.50)	(35.00)	(31.80)	(47.20)	(31.10)	(32.00)	(47.70)	(28.30)
Proportion scheduled tribe HHs in district	−14.50	−26.20	1.96	11.80	25.40	−5.14	12.40	26.40	−5.02
	(13.80)	(23.00)	(12.10)	(13.60)	(22.40)	(10.70)	(13.70)	(22.70)	(9.73)
Proportion other backward class HHs in district	11.50	16.20	22.50	−7.79	−6.81	22.50	−7.80	−4.56	21.10
	(14.10)	(21.30)	(15.90)	(14.20)	(21.60)	(14.10)	(14.30)	(22.00)	(12.80)
Proportion of HHs living in urban area in district	−14.80	−56.00	31.60	−25.30	−77.5*	20.10	−19.10	−68.2*	15.50
	(18.90)	(29.90)	(19.90)	(19.20)	(30.60)	(17.70)	(19.40)	(31.30)	(16.10)
Mean maternal age	3.98	4.22	0.20	4.61*	3.85	−0.59	5.00*	3.69	0.01
	(2.16)	(3.63)	(2.54)	(2.18)	(3.70)	(2.25)	(2.19)	(3.76)	(2.05)
Mean parity	−3.78	−1.36	9.71	−0.76	27.50	9.80	−4.65	19.30	7.38
	(5.37)	(13.90)	(5.87)	(5.47)	(14.10)	(5.14)	(5.52)	(14.40)	(4.68)
Mean maternal education	3.07*	3.56	−0.89	1.04	−2.78	−0.74	2.48	−0.54	−0.36
	(1.52)	(2.76)	(2.21)	(1.56)	(2.84)	(1.96)	(1.57)	(2.89)	(1.78)
Mean # ANC visits	5.53***	7.21**	−3.40	3.56*	1.45	−3.36	3.13	0.00	−3.45
	(1.60)	(2.70)	(4.72)	(1.68)	(2.90)	(4.19)	(1.70)	(2.95)	(3.80)
Mean receiving all DPT vaccinations	19.80	12.30	−3.06	7.06	−45.30	−5.29	1.97	−62.20	−9.86
	(16.90)	(34.80)	(18.10)	(17.20)	(35.00)	(16.00)	(17.40)	(35.20)	(14.50)
Logged state domestic product	−4.34	−8.38	8.44*	−11.3***	−16.9***	9.44**	−12.9***	−18.5***	9.36**
	(3.23)	(4.50)	(4.11)	(3.10)	(4.27)	(3.62)	(3.06)	(4.21)	(3.27)

Notes: Constants estimated but not reported. Robust standard errors in parentheses. * p < 0.05, ** p < 0.01, *** p < 0.001.

## Results

### Rising food prices and child survival across Indian districts

Supplementary Figure 1 shows the percentage change in all food prices between 2002 and 2008, adjusted for inflation, in India’s 364 districts for which comparative price data were available. Total food prices across food items began rising in 2004, with the exception of a modest drop in 2006–07. The greatest increase in prices overall occurred in the 2007–08 period. Across a background of rising total real food prices, there were marked variations by the type of food. For example, between 2002 and 2008, the peak annual price rises in any year for pulses and dairy respectively were 29.8% and 27.0%. The geometric mean of the price change across the entire 6-year period was 36.1% for pulses and 37.3% for dairy. Peak rises in meat prices were also substantial but smaller, with the peak annual price rise of 13.9% occurring between 2007 and 2008. However, the geometric mean rise across the entire period, 36.1%, was similar to that of pulses and dairy. Price rises were sharper and more volatile in EAG than in non-EAG states, with a peak rise of 13.4% in EAG states in 2007–08 compared with a peak of 8.9% in non-EAG states in the same period. However, the geometric mean rise in all prices across the 6-year period was quite similar, at 35.9 % in EAG states compared with 35.6% in non-EAG states.

There was marked variation in the change in both price fluctuations and mortality rates across districts. Supplementary Figures 2 and Supplementary Data (available as Supplementary data at *IJE* online) show the percentage change in food prices and mortality, respectively, between 2002 and 2008. Price and mortality fluctuations were highly uneven between districts, with some districts experiencing a decline whereas others experienced an increase. In many districts, price rises were substantial, as much as 77% over the 6-year period. An increase of more than 30% was observed in 21 districts. Although most districts experienced a modest decline in mortality, in some there were sharp increases. These cross-district price variations provide an opportunity to investigate the link between food prices and child mortality.

### Association of food price rises with child survival

Supplementary Table 1 (available as Supplementary data at *IJE* online) presents the full set of results for the annual difference models of the relation between changes in real food prices averaged across all food items for NNMR, IMR and U5MR, adjusting poverty rates, maternal age and education, state domestic product and other potential socio-demographic confounding factors. We found that a 1% increase in total food price of all food items combined was associated with a 0.49% increase in NNMR (95% CI: 0.13% to 0.85%, *P* = 0.01). There was only weak evidence of an association with IMR (b = 0.31; 95% CI: -0.01% to 0.64%) and U5MR (b = 0.16%; 95% CI: -0.14% to 0.47%). Consistent with previous research, we observed that the change in child mortality rates was lower in areas that were more urbanized and had a lower proportion of persons who were backward caste. As expected, we also found a negative association between the change in state domestic product and the change in mortality.

**Figure 1.**
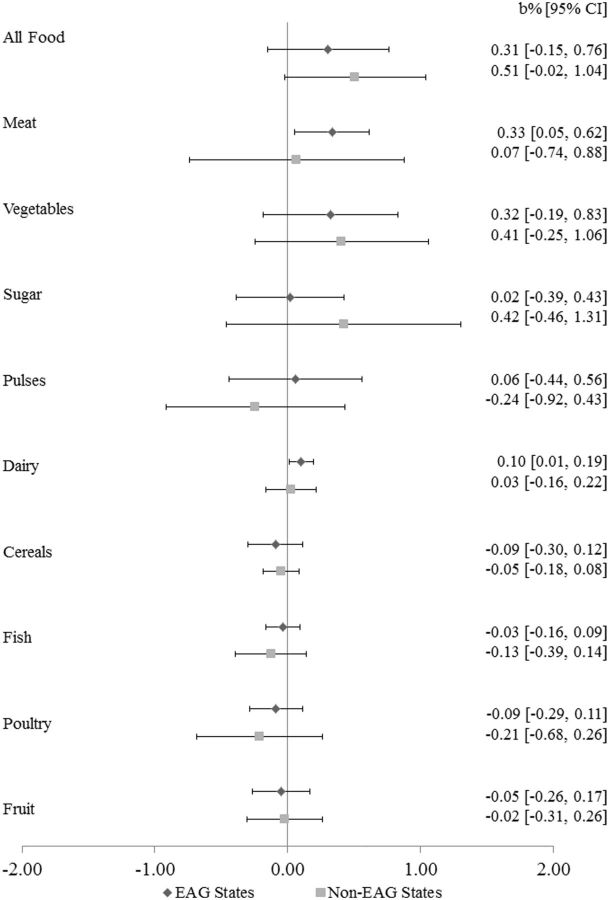
Association of a 1% change in food prices with percent change in neonatal mortality rates, 35 states and territories.

**Figure 2.**
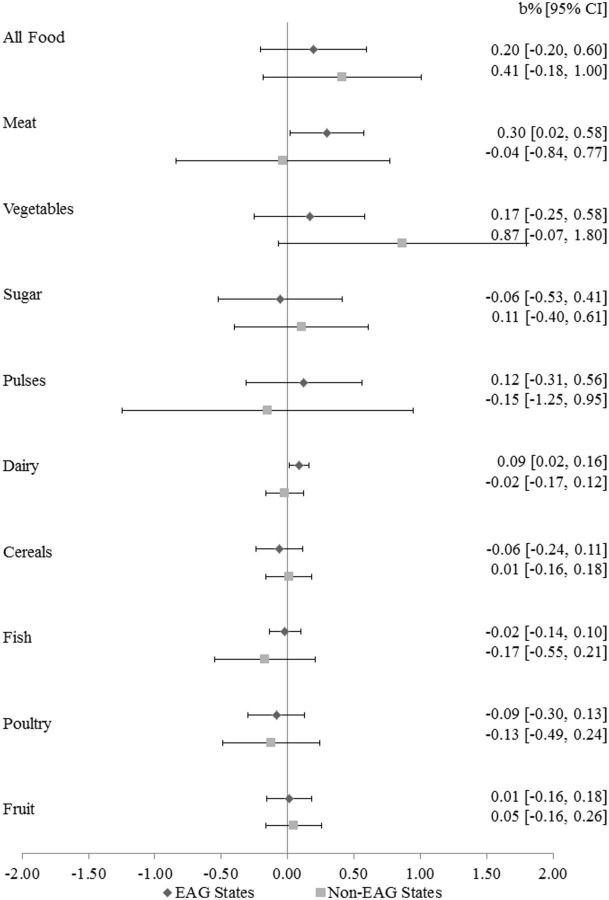
Association of a 1% change in food prices with percent change in infant mortality rates, 35 states and territories.

**Figure 3.**
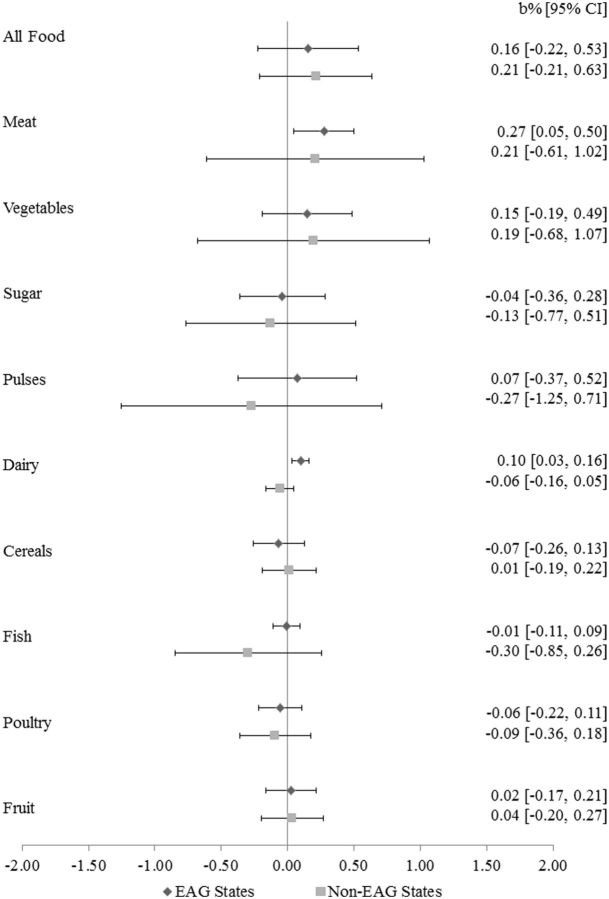
Association of a 1% change in food prices with percent change in under-five mortality rates, 35 states and territories.

Next we disaggregated the sample into deprived (EAG) and less-deprived (non-EAG) states, testing the hypothesis that the harms from food price rises would be greater in economically deprived regions where households were already more food insecure. Figure 1 shows a forest plot of 20 statistical models, covering 10 food categories. In EAG states, we observed a positive association of meat and dairy prices with NNMR. Specifically, we found that with each 1% increase in the prices of meat there was a 0.33% increase (b = 0.33%; 95% CI: 0.05% to 0.62%; *P* = 0.03), and with a 1% increase in the prices of dairy there was a 0.10% increase (b = 0.10%; 95% CI: 0.01% to 0.19%; *P* = 0.01) in the percentage change in NNMR. In non-EAG states, there was no association between rising food prices and changes in NNMR. Figures 2 and 3 extend the models to the change in infant and under-five mortality rates, respectively. Similar patterns were observed. In EAG states, each 1% rise in dairy prices was associated with a 0.09% increase in infant mortality rates (95% CI: 0.02% to 0.16%; *P* = 0.02) and a 0.10% increase in under-five mortality (95% CI: 0.03% to 0.16%; *P* = 0.01). Similarly, a 1% increase in meat prices was associated with a 0.30% (95% CI: 0.02% to 0.58%; *P* = 0.04) increase in the IMR and a 0.27% increase in the U5MR (95% CI: 0.05% to 0.50%; *P* = 0.03). In the less deprived (non-EAG) states, we did not observe adverse associations of any of the food categories with either IMR or NNMR.

### Robustness tests

We performed several robustness checks. First, we tested adjusted inference testing for multiple comparisons across food items. We applied a Liu correction, a false discovery rate adjustment used when the number of comparisons is relatively small. ^35^ As shown in the smileplots in Appendix Figures W1–W3 (available as Supplementary data at *IJE* online) which plot *P* -values on a reverse log scale against parameter estimates, our main results remain unchanged. Next, to test further potential confounding due to quality and utilization of the healthcare system, we controlled for the total average number of DPT immunizations and the proportion of mothers with antenatal care access. None of the results was changed. Since gross state domestic product could positively associate with food price inflation and so attenuate results, we replicated models without these adjustments. Although some associations were slightly strengthened, differences were modest. Finally, in view of the importance of iron and folic acid supplementation in preventing NNMR, we replicated models including the mean district-level uptake of iron and folic acid supplementation. None of the results was substantially altered.

## Discussion

This study investigated the association between food prices and child mortality in India at the district level. The substantial variation in food prices across districts created a unique opportunity to identify how food price changes impact on child survival. Our study reveals three important findings. First, the recent rises in food prices in India are associated with worse neonatal, infant and under-five mortality rates. Second, these adverse associations were concentrated in India’s economically deprived states, which have a higher burden of malnutrition. Third, of the multiple food items tested, price rises in high-protein foods, namely meat and dairy products, conferred the greatest magnitude of mortality risk to children. Fourth, the risk of mortality associated with rising food prices appears to be greatest for neonates.

Our study has several limitations. First, as with all district-level analyses, there is potential for ecological fallacy. However, the relationships tested are biologically plausible and consistent with a large body of econometric evidence indicating that price rises correspond to declines in consumption of food staples. ^47^^,^^48^ Even so, we are not able to assess causality with the available data; additional research is needed to explore further the links between food prices and child mortality. Second, the most recent available data at the time of study were from 2008, before the time of the global financial crisis. This would not confound our study, but future research is needed to evaluate the harms that may have resulted from sharp price rises associated with the global recession. Third, several districts had missing data on food prices, reflecting how collection is randomly conducted at local market hubs. However, since this missingness was by definition random, it is unlikely to bias systematically any findings.

Additionally, data were unavailable to track the actual consumption of specific food items over time. This meant we were unable to assess potential substitution effects that occur when food prices increase. For example, as the price of meat rises, households may consume lower-cost foods, such as cereals and grains. Future research is also needed to investigate the role of relative price changes. Households could substitute within food categories (such as switching from higher cost/quality rice to lower cost/quality rice) or between categories. Our broad food categories were unable to distinguish the former possibility, and including both real and relative price shifts was not feasible due to high multi-collinearity upon inclusion of both sets of factors. Nonetheless, in a context where real food prices are rising markedly, these increases are likely to be the dominant factor in shifting the overall quantity of household food choices. As with all studies using DLHS data, wage data are unavailable, reflecting the importance of informal economy; our study adjusted for a household wealth index. Nonetheless, to address the possibility that higher food prices are simply correlated with a higher standard of living, we adjusted all prices for inflation using the consumer price index for non-food items. Future research is needed to understand how household resilience and coping strategies may exacerbate or mitigate the nutritional consequences of food price rises. However, the coping strategies used during this time appear to have been ineffective in promoting child survival. Finally, although we controlled for mean number of ANC visits and mean number of children receiving all recommended DPT vaccinations as a proxy for quality of and access to the healthcare system, it is possible that other health system factors (e.g. institutional deliveries) could relate to both mortality outcomes and food prices. Further investigation into the role of these factors is needed.

There are several underlying pathways which plausibly link food price fluctuations to child survival, particularly concentrated in the neonatal period, as observed here. First, and most obviously, food prices may shape dietary intake for both mothers, whose nutrition is vital for prenatal development ^36^ and production of breast milk, ^37^^,^^38^ and for children, who should begin complementary consumption of solid, semi-solid and soft foods beginning at 6 months of age in order to reach caloric adequacy and avoid micronutrient deficiencies. ^39^ Second, intrahousehold dynamics may shape resilience strategies in the face of rising food prices, with important implications for both maternal and child health. Evidence from Indonesia, ^40^ for example, suggests that when households face food insecurity, mothers will forgo calories in order to ensure the needs of their children are met, resulting in stagnation of child nutrition paired with increased maternal wasting in times of scarcity. Thus, mothers rather than young children may bear the brunt of food insecurity. Given the importance of maternal nutrition for perinatal outcomes, ^41–43^ poor maternal nutrition in this context may help to explain higher neonatal but not child mortality concomitant with rising food prices. Future research is needed to investigate these mechanisms.

It is important for future research to test the potential of India’s Food Security Programmes, primarily the Public Distribution System and the Midday Meal Scheme, to mitigate risks to child health from food price rises. Some studies have suggested that such programmes are either ineffective or in fact contribute to worse child health outcomes, ^44–46^ but further research using individual-level longitudinal data is needed. These programmes may be maximally effective during periods of food hardship.

Our findings advance on a large body of work on malnutrition and child survival by looking at the role of a major underlying economic determinant: the cost of food. Previous cross-sectional, comparative work has found that malnutrition is a substantial driver of child mortality ^13–15^ and multiple randomized controlled trials (RCTs) have demonstrated the particular importance of micronutrient and caloric deficiencies in increasing malnutrition risk ^49–54^ and child mortality. ^55^^,^^56^ Whereas studies have suggested that rising prices may adversely impact on food security and nutrition, ^8^ the available research has sought to link conceptually food prices and nutrition ^40^^,^^57^^,^^58^ or nutrition and survival, ^13–15^^,^^59^ without directly assessing the association between rising prices and child mortality outcomes. Another strand has been based on simulation studies, lacking empirically tested data. ^27^^,^^60^ To our knowledge, the only study investigating this topic directly was primarily a descriptive documentation of trends in both food prices and child health over time, and the study did not include India ^19^ where rates of child malnutrition are especially high. ^5^ Here we document empirical evidence that rising food prices are negatively associated with child survival in India. The continued rise in food prices in recent years, projected to rise further in the coming decade, ^61^ is likely to contribute further to slow progress towards the Millennium Development Goals (or Sustainable Development Goals as of 2016) for childhood survival. ^22^

Our findings have important policy implications. High-protein foods, particularly meat and dairy, appear to be protective factors in Indian children’s diets. We observed wide fluctuations in food prices across districts; even within states, food prices rise in some areas while falling in others. Future research is needed to identify factors which may alleviate both food price escalation and its health consequences. One example is the Public Distribution System, which subsidizes staple foods (rice, sugar, pulses). At present, however, these programmes exclude high-protein meat and dairy products, food categories most strongly correlated with child mortality. Food price rises appeared to most greatly affect economically disadvantaged states, suggesting that current food subsidy policies may not be sufficient in the context of decreasing food affordability. India’s food security programmes may need to cover additional components of healthy diets. Whereas there has been extensive focus on micronutrient supplementation, the potential gains from these interventions may not be fully realized if food prices continue to escalate.

In conclusion, India’s rising food prices, particularly of high-protein meat and dairy products, were associated with worse child mortality outcomes. These potentially avoidable adverse associations were concentrated in India’s eight most deprived states.

## Author Contributions

J.F., S.E., and D.S. conceptualized the study; J.F., S.V. and Z.K. conducted the analysis; J.F., S.V., Z.K., S.E and D.S. contributed to interpretation of the results; J.F. and D.S. drafted the manuscript; all authors contributed to revision of the manuscript and approved the final version.

### Supplementary Data

Supplementary data are available at *IJE* online.

## Supplementary Material

Supplementary Data
