# Supplementary material for: Quantifying the impact of rising food prices on child mortality in India: a cross-district statistical analysis of the District Level Household Survey
Source: Int J Epidemiol. 2016 Apr 10;45(2):554–64. doi: 10.1093/ije/dyv359 (PMC4864878; doi:10.1093/ije/dyv359)
Supplement: Supplementary Data [file dyv359_supplementary_data.zip › ije-2015-06-0750-File002.docx]

Web Table I. Regression of % Change in All Food Prices on % Change in Mortality, Full Models

|  | Model 1 | Model 2 | Model 3 |
| --- | --- | --- | --- |
|  | Percentage change in NNMR | Percentage change in IMR | Percentage change in U5MR |
| Percentage change in food prices | 0.49^**^ | 0.31 | 0.16 |
|  | (0.17) | (0.16) | (0.15) |
| Proportion of Hindu HHs in district | 12.10 | -13.40 | 3.74 |
|  | (19.60) | (23.50) | (24.60) |
| Proportion of Muslim HHs in district | 124.4^***^ | 98.3^**^ | 61.10 |
|  | (25.30) | (31.30) | (29.80) |
| Proportion scheduled caste HHs in district | 55.90 | 46.60 | 6.90 |
|  | (51.70) | (40.90) | (34.80) |
| Proportion scheduled tribe HHs in district | 33.80 | 32.20 | 19.30 |
|  | (24.90) | (24.00) | (19.10) |
| Proportion scheduled other backward class HHs in district | 39.0^*^ | 35.90 | 33.50 |
|  | (15.20) | (18.40) | (17.30) |
| Proportion of HHs living in urban area in district | -37.90 | -25.40 | -25.60 |
|  | (22.30) | (16.80) | (16.90) |
| Mean maternal age | -0.54 | 4.22 | 1.48 |
|  | (3.38) | (4.47) | (3.68) |
| Mean parity | 1.52 | -7.36 | -13.20 |
|  | (8.48) | (10.80) | (9.86) |
| Mean maternal education | 3.19 | -2.91 | -0.16 |
|  | (2.42) | (4.51) | (2.82) |
| Mean Number of ANC visits | 5.31^*^ | 3.54 | 3.00 |
|  | (2.26) | (3.30) | (3.26) |
| Mean receiving all DPT vaccinations | 21.40 | 15.70 | 3.93 |
|  | (24.40) | (23.40) | (21.20) |
| Logged state domestic product | -6.50 | -9.99^*^ | -12.5^**^ |
|  | (4.02) | (3.66) | (3.49) |
| Percentage change in state domestic product | -109.3^*^ | -117.2^*^ | -165.5^*^ |
|  | (45.20) | (53.80) | (76.30) |
| *R*^2^ | 0.18 | 0.17 | 0.14 |

Notes: Constant estimated but not reported. Robust standard errors in parentheses.

* p < 0.05, ** p < 0.01, *** p < 0.001
