# Supplementary figures and images for: Quantifying the impact of rising food prices on child mortality in India: a cross-district statistical analysis of the District Level Household Survey
Source: Int J Epidemiol. 2016 Apr 10;45(2):554–64. doi: 10.1093/ije/dyv359 (PMC4864878; doi:10.1093/ije/dyv359)

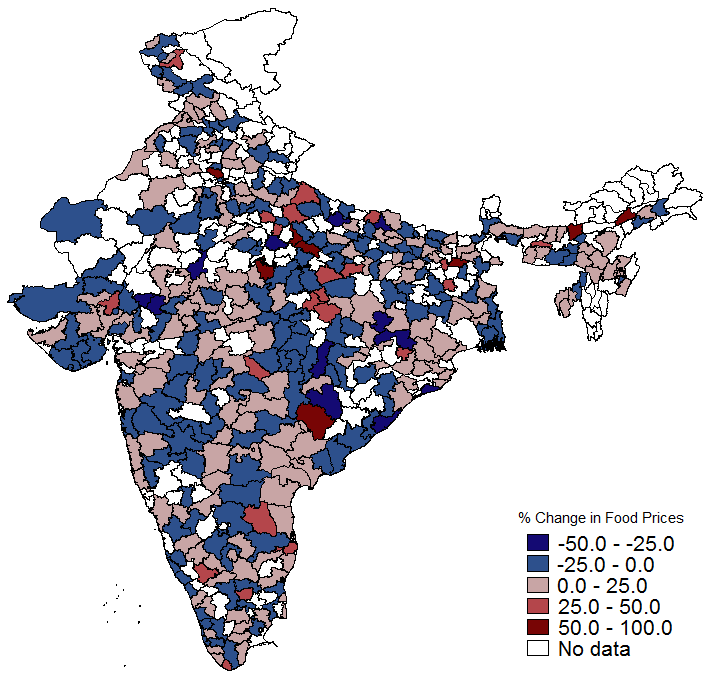

Supplement: Supplementary Data [file dyv359_supplementary_data.zip › ije-2015-06-0750-File008.tif]

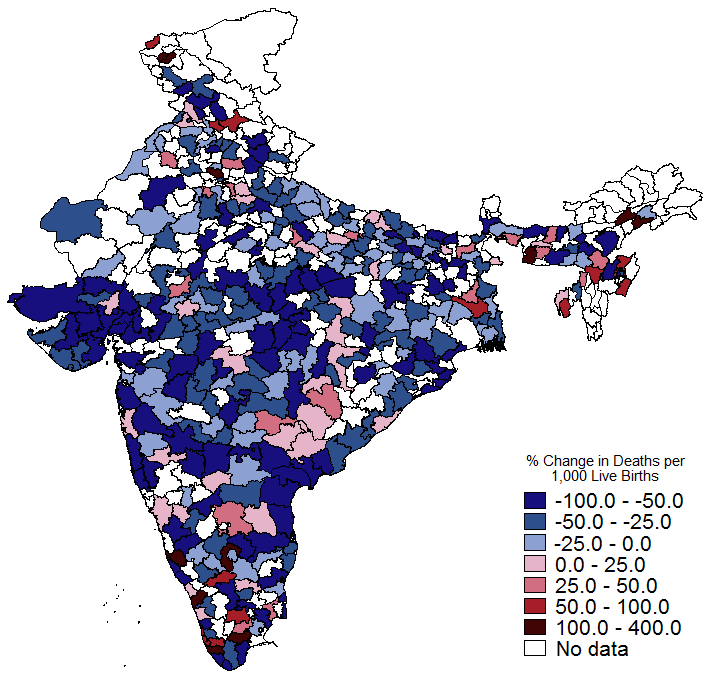

Supplement: Supplementary Data [file dyv359_supplementary_data.zip › ije-2015-06-0750-File009.tif]

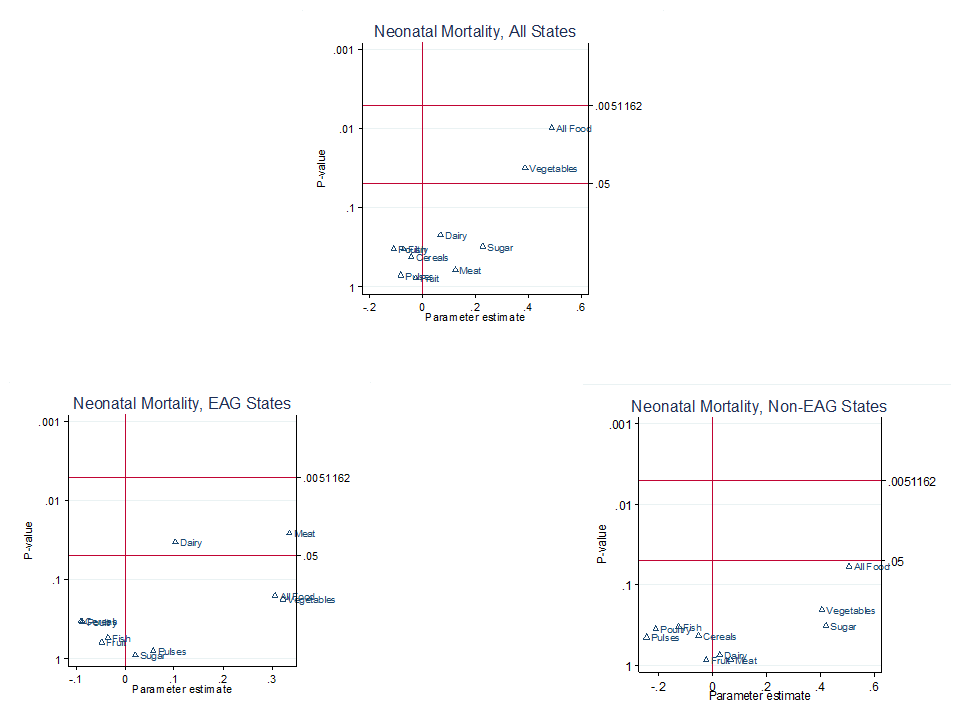

Supplement: Supplementary Data [file dyv359_supplementary_data.zip › ije-2015-06-0750-File010.tif]

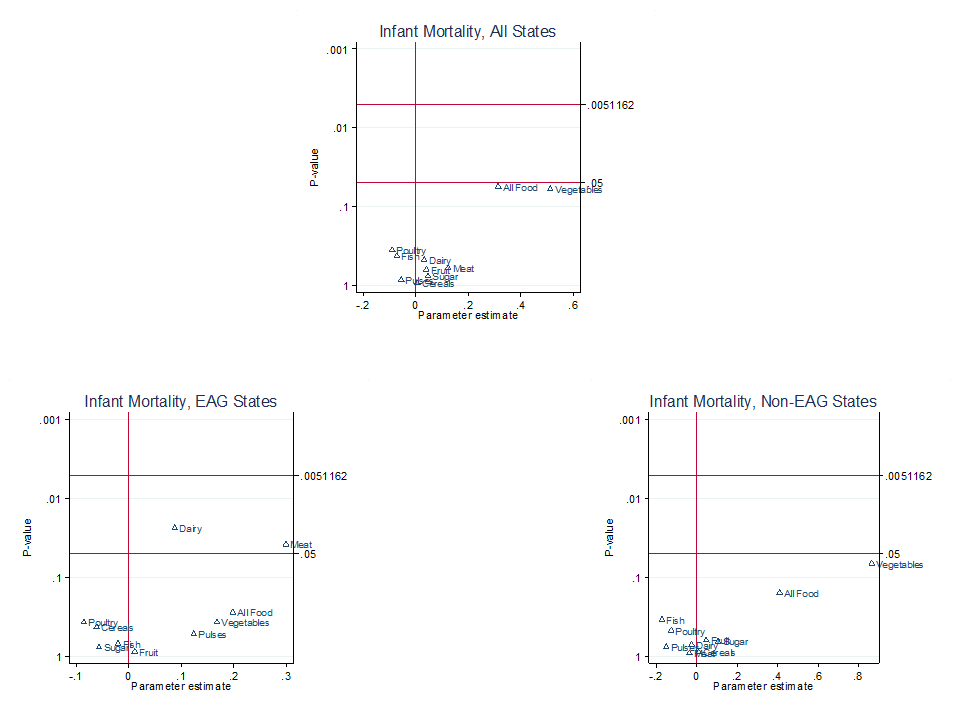

Supplement: Supplementary Data [file dyv359_supplementary_data.zip › ije-2015-06-0750-File011.tif]

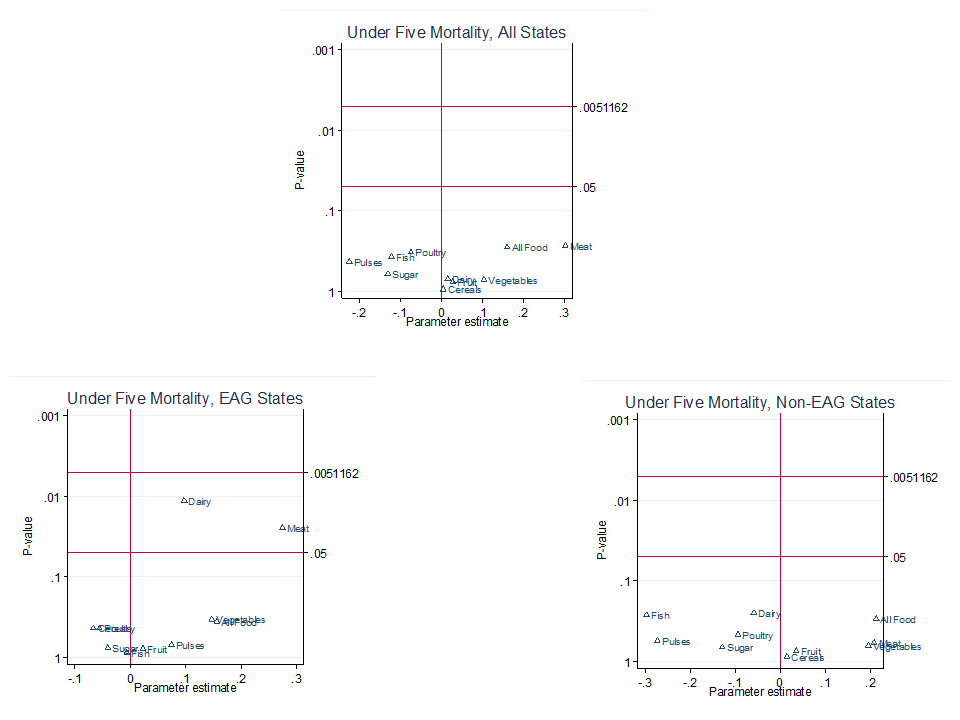

Supplement: Supplementary Data [file dyv359_supplementary_data.zip › ije-2015-06-0750-File012.tif]

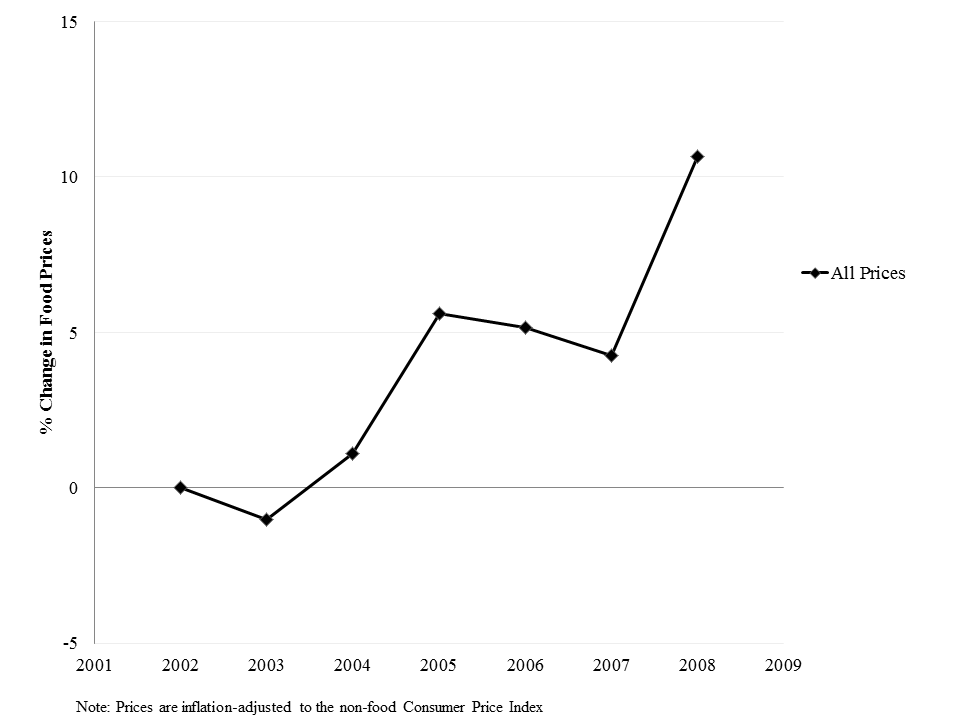

Supplement: Supplementary Data [file dyv359_supplementary_data.zip › ije-2015-06-0750-File007.tif]
